# Factors Associated With Intention to Use Internet-Based Testing for Sexually Transmitted Infections Among Men Who Have Sex With Men

**DOI:** 10.2196/jmir.2888

**Published:** 2013-11-14

**Authors:** Mark Gilbert, Travis Salway Hottes, Thomas Kerr, Darlene Taylor, Christopher K Fairley, Richard Lester, Tom Wong, Terry Trussler, Rick Marchand, Jean Shoveller, Gina Ogilvie

**Affiliations:** ^1^Clinical Prevention ServicesBC Centre for Disease ControlVancouver, BCCanada; ^2^School of Population and Public HealthUniversity of British ColumbiaVancouver, BCCanada; ^3^Department of MedicineUniversity of British ColumbiaVancouver, BCCanada; ^4^BC Centre for Excellence in HIV/AIDSVancouver, BCCanada; ^5^Melbourne School of Population and Global HealthUniversity of MelbourneVictoriaAustralia; ^6^Public Health Agency of CanadaOttawa, ONCanada; ^7^Community Based Research Centre for Gay Men's HealthVancouver, BCCanada

**Keywords:** homosexuality, male, Internet, testing, human immunodeficiency virus, sexually transmitted infection, health equity, patient acceptance of health care

## Abstract

**Background:**

Internet-based testing programs are being increasingly used to reduce testing barriers for individuals at higher risk of infection, yet the population impact and potential for exacerbation of existing health inequities of these programs are not well understood.

**Objective:**

We used a large online sample of men who have sex with men (MSM) in Canada to measure acceptability of Internet-based testing and perceived advantages and disadvantages of this testing approach.

**Methods:**

We asked participants of the 2011/2012 Sex Now Survey (a serial online survey of gay and bisexual men in Canada) whether they intended to use Internet-based testing and their perceived benefits and disadvantages of use. We examined whether intention to use was associated with explanatory variables spanning (A) sociodemographics, (B) Internet and technology usage, (C) sexually transmitted infections (STI)/ human immunodeficiency virus (HIV) and risk, and (D) health care access and testing, using multivariable logistic regression (variable selection using Bayesian information criterion).

**Results:**

Overall, intention to use was high (5678/7938, 71.53%) among participants with little variation by participant characteristics. In our final model, we retained the variables related to (B) Internet and technology usage: use of Internet to cruise for sex partners (adjusted odds ratio [AOR] 1.46, 95% CI 1.25-1.70), use of Internet to search for sexual health information (AOR 1.36, 95% CI 1.23-1.51), and mobile phone usage (AOR 1.19, 95% 1.13-1.24). We also retained the variables for (D) health care access and testing: not “out” to primary care provider (AOR 1.24, 95% CI 1.10-1.41), delayed/avoided testing due to privacy concerns (AOR 1.77, 95% CI 1.49-2.11), and delayed/avoided testing due to access issues (AOR 1.65, 95% CI 1.40-1.95). Finally, we retained the variable being HIV positive (AOR 0.56, 95% CI 0.46-0.68) or HIV status unknown (AOR 0.89, 95% CI 0.77-1.01), age <30 years (AOR 1.41, 95% CI 1.22-1.62), and identifying as bisexual (AOR 1.18, 95% CI 1.04-1.34) or straight/other (AOR 0.67, 95% CI 0.50-0.90). The greatest perceived benefits of Internet-based testing were privacy (2249/8388, 26.81%), general convenience (1701/8388, 20.28%), and being able to test at any time (1048/8388, 12.49%). The greatest perceived drawbacks were the inability to see a doctor or nurse (1507/8388, 17.97%), wanting to talk to someone about results (1430/8388, 17.97%), not wanting online results (1084/8388, 12.92%), and low trust (973/8388, 11.60%).

**Conclusions:**

The high and wide-ranging intention to use that we observed suggests Internet-based testing has the potential to reach into all subgroups of MSM and may be particularly appealing to those facing current barriers to accessing STI/HIV testing and who are more comfortable with technology. These findings will be used to inform the promotion and further evaluation of an Internet-based testing program currently under development in British Columbia, Canada.

## Introduction

Public health agencies are increasingly turning to the Internet for the delivery of sexual health services in order to reduce barriers to access and reach people at heightened risk of sexually transmitted infections (STI) [1-3]. Internet-based testing reflects a recent and fundamental shift in delivery of testing services, from provider-mediated to patient-centered testing [4-6], with the aim of reducing barriers to accessing traditional testing services (such as needing to travel to a clinic or waiting for an appointment, or privacy and confidentiality concerns). Internet-based testing programs typically involve requesting a home self-collection kit or downloading a test requisition and presenting it to a specimen collection site, then receiving results online or by phone. The scope of public programs varies widely, from state or country-wide programs for chlamydia screening [7-9], to more local programs that offer one or more STI/ human immunodeficiency virus (HIV) tests and may be targeted to a specific population such as gay, bisexual, and other men who have sex with men (MSM) [10,11].

The evidence of the impact of Internet-based testing programs is beginning to accumulate, primarily for population-based chlamydia screening programs [8,12,13]. However, substantial knowledge gaps remain regarding the impact of these services at a population level, such as the reach and diffusion of programs within populations at higher risk for STI/HIV [14]. An important concern with the introduction of new health technologies is that uptake may be concentrated among individuals who already have good access to health services (often correlated with socioeconomic status) and not among individuals who need it most [15,16]. For example, if the uptake of Internet testing programs is highest among individuals who already have adequate access to testing, these programs may run the risk of reinforcing rather than reducing health inequities if not accessed by individuals currently facing barriers to testing. The alluring promise of, but widespread lack of delivery by, online technologies to expand health and health access to larger portions of the population, particularly the more marginalized, is a topic of concern [17-21]. This concept applies not just to, but within, marginalized populations such as MSM where both sexual risk, disease prevalence, and appropriate access to health care are unevenly distributed [22].

Internet-based interventions are widely recognized as a valuable tool for promoting sexual health among MSM [23]. MSM have a high burden of STIs and HIV in Canada, as well as in most industrialized countries. MSM comprise approximately 50% of all incident HIV and prevalent infections in Canada [24] with high rates of other STIs including ongoing syphilis outbreaks in several provinces and countries with comparable STI epidemiology [25]. MSM are also likely to turn to the Internet to look for sexual health information or support, and finding sex partners through sex-seeking websites is widespread, including by men at greater sexual risk of infection [23,26]. Studies of Internet-based testing programs have demonstrated that significant numbers of MSM use these services [16,18]. However, few studies assessing the reach or broader acceptability of Internet-based testing among MSM have been published [4]. Studies have examined the willingness of MSM to access anonymous home HIV testing as part of online research [27] and the acceptability of other online interventions such as partner notification [28-31]; overall, acceptability is high with small but significant differences across subgroups. Factors associated with acceptability or uptake of Internet-based sexual health services (in males, MSM or youth) include income [32], age [31,32], ethnicity [27,31], education [31], substance use [32,33], HIV status [27,29,33], prior STI [29,33], risk sex [27,31,33-35], perceived risk of HIV [34], and health seeking behavior [36]. Men living in rural areas may be more likely to find sex partners online and be willing to participate in online interventions [36-38]. There is little discussion of potential harms of online sexual health interventions in general or for MSM in particular. The main concerns relate to their inaccessibility by people with no or limited Internet access (the so-called “first-level” digital divide) or by people who have less facility with using the Internet (the “second-level” digital divide) [39]. Recently, Rosser et al emphasized the importance of considering how different age groups of MSM approach technology and use the Internet in the design of Internet-based sexual health services [40]; however, the impact of technology use on acceptability of online services is largely unknown.

The British Columbia (BC) Centre for Disease Control is developing a program, GetCheckedOnline, for Internet-based testing for STIs and HIV and is planning a targeted promotion of the service to MSM in the Vancouver area. The program has been developed through focus groups with MSM and youth who have indicated high acceptability of the service [41,42] and if successful, the intent is to expand the program on a broader geographic scale. The primary objective of this study was to assess the acceptability of Internet-based testing in a national sample of MSM (based on intention to use) and associated characteristics. In so doing, we aimed to assess acceptability and potential reach of Internet-based testing among MSM with varying sociodemographic characteristics, with greater reported risk of infection for STI and HIV, and facing existing barriers to testing access. A secondary objective of the study was to describe the perceived advantages and disadvantages of Internet-based testing. By identifying factors associated with intention to use and perceptions of Internet-based testing prior to implementation of the program, we will then be better positioned to further refine the service, target its promotion to particular subgroups, and develop strategies to promote acceptance and adoption of the service [43].

## Methods

### Survey

Sex Now is a national online survey of gay and bisexual men, administered every 12-18 months in Canada [44]. Sex Now content is developed iteratively by a panel of gay men’s health researchers, with the aim of responding to evolving needs of the community. Face validity of the questionnaire is ensured through focus groups, interviews, and pilot testing by local gay men. Standard survey domains include relationships, sexual styles, sexual behaviors, sexual health, anti-gay or discriminatory experiences (especially in workplace), substance use, sexual health knowledge, Internet experience, health care access, community participation, mental health issues, and sociodemographics. Questions are available in both French and English.

Participants from the 2011-12 cycle were recruited through dating/sex-seeking websites (6356/8388, 75.8%), gay/bisexual community-based organizations (833/8388, 10.0%), and word-of-mouth (729/8388, 8.7%). The survey is described as a survey of “sex between men” and relies on self-selection. Responses were collected from August 26, 2011, to February 21, 2012. To limit multiple entries, submissions were restricted to one response per Internet Protocol address, and data were rigorously examined to screen out multiple or suspect submissions.

A subset of questions relevant to the BC Internet-based STI/HIV testing model were added to the 2011-12 questionnaire for the purposes of the present study. The following question domains were added: barriers to clinic-based testing; acceptability of Internet-based testing; perceived benefits, risks, and barriers of Internet-testing; and potential factors influencing uptake of Internet-based testing including privacy concerns, use of health services, and use of technology. We used the concept of intention or willingness to use the technology, which is an established metric for measuring acceptance of technology and considered predictive of actual use in theoretical models of technology acceptance [43,45]. As we were also interested in informing strategies for acceptance and adoption, we considered diffusions of innovations theory [46,47] and use of online/mobile technologies to potentially be important predictors of intention to use Internet-based testing among MSM, based on evidence emerging from recent analyses of online sex-seeking behavior [40]. Due to a lack of established metrics to measure these latter concepts, we constructed questions that were validated through the process described above. For all variable definitions, see Multimedia Appendix 1.

### Measures

The primary outcome of interest was intention to use Internet-based STI/HIV testing, measured through a 5-point Likert scale response to the following question: “Suppose you could get tested by printing out an order form from a website that you could take to a lab, then get your results online. How likely is it that you would use this service? [Very likely, likely, unlikely, very unlikely, would never use this service; with ‘not applicable’ option]”. Participants were also asked to identify a single greatest perceived benefit and drawback of the aforementioned service from a pre-determined list. Response options were determined based on expert knowledge of local STI clinical services and literature describing other Internet-based STI testing models.

### Analysis

The sample was restricted to participants residing in Canada (8388/8497, 98.72%), of which 94.64% (7938/8388) completed the question on intention to use Internet-based testing. All respondents were male. Thirty-eight explanatory variables were selected from the questionnaire a priori based on the literature review described above and were grouped into four broad groups of interest: (A) sociodemographics (14 variables), (B) Internet and technology usage (6 variables), (C) STI/HIV and risk (8 variables), and (D) health care access and testing (10 variables).

To achieve the primary objective of identifying characteristics correlated with intention to use Internet-based STI/HIV testing, we first explored the distribution of responses (5-point Likert scale) across all 38 explanatory variables. Logistic regression was then used to model associations between all explanatory variables and this outcome (dichotomized: very likely/likely versus unlikely/very unlikely/never; those who chose “not applicable” were excluded). A full multivariable model was fit with all 38 explanatory variables, and a final model was selected using Bayesian information criterion (BIC), which is comparable to Akaike information criterion (AIC) but imposes stricter penalties for inclusion of additional explanatory variables (ie, generates a more parsimonious model) [48]. Correlation between explanatory variables was examined, and the covariate considered most relevant to the research question was included in multivariable models for highly collinear sets.

Age groups (less than 30 years, 30 years of age and older) and sexual orientation (gay, bisexual) were identified a priori as subgroups of interest and hence were included in all multivariable models. We hypothesized that other explanatory variables would differ across these subgroups and explored statistical interactions between explanatory variables and age and sexual orientation. Each variable was entered into two bivariable models, including age and sexual orientation respectively. First-level multiplicative interaction terms were added, and interaction terms that were statistically significant at *P*<.10 were carried forward in analysis. Stepwise regression was used to select interaction terms for inclusion in the full multivariable model, such that all remaining interaction terms were significant at *P*<.15 [49].

To achieve the secondary objective, perceived benefits and drawbacks of the service were summarized using descriptive statistics for the total sample, and among men who delayed or avoided STI or HIV testing in the past 12 months (STI testing only if HIV positive).

All analysis was completed using R version 2.15.2. BIC model selection was performed using the stepAIC function in MASS package version 7.3-22 [50].

### Ethics Approval

The survey protocol was approved by the independent Research Ethics Board of the Community-Based Research Centre and also by the Behavioural Research Ethics Board at the University of British Columbia.

## Results

### Summary of Sample

Characteristics of the sample are shown in Multimedia Appendix 2. The average age was 43 years (range 13-84 years), and 64.50% (5410/8388) self-identified as gay and 32.42% (2719/8388) as bisexual. Further, 57.13% (4792/8388) had completed a college or university degree, and 71.46% (5994/8388) reported annual incomes ≥ Canadian $30K. The sample was predominantly urban (4897/8388, 58.38%), though a significant proportion resided in suburban (2214/8388, 26.39%) or rural/remote (1245/8388, 14.84%) settings. Respondents included residents of all ten Canadian provinces and all three territories, with the distribution generally representative of total regional populations with the exception of British Columbia (greater proportion) and Quebec (smaller proportion) [51]. Respondents represent 71.61% (1173/1638) of the forward sortation areas (first three characters of the postal code) of Canada [52]. Most men reported being “out” about their sexuality generally (5295/8388, 63.13%), but fewer were out at work (3881/8388, 46.27%), and fewer still spent most of their free time with other gay men (1867/8388, 22.26%).

### Intention to Use Internet-Based STI/HIV Testing

Of the total sample, 71.53% (5678/7938) indicated that they were likely (2422/7938, 30.51%) or very likely (3256/7938, 41.02%) to use Internet-based STI/HIV testing. Across the full 5-point response scale, intention to use Internet-based testing was right-skewed towards very likely to use, with little variation across subgroups (data not shown). Dichotomized intention to use Internet-based testing was similarly high across nearly all covariates examined, generally ranging 67-77%, with few exceptions: Latino men (88/109, 80.7%), very early purchasers of new technology (270/338, 79.9%), those not at all satisfied with health care services (290/361, 80.3%), and those who delayed or avoided testing in the past 12 months for privacy concerns (944/1128, 83.69%), access issues (1021/1225, 83.35%), or distance from clinic (357/429, 83.2%) (see Multimedia Appendix 2). Intention to use Internet-based testing was lower than 67% in only two subgroups: HIV-positive men (263/476, 55.3%) and regular users of party drugs (105/159, 66.0%).

Crude and adjusted odds ratios (AOR) are shown in Multimedia Appendix 2. In the full model, the following explanatory variables retained statistical (*P*<.05) associations with greater intention to use Internet-based testing: group A (3/14 variables), age <30 years, eastern provinces, less “out” about sexuality at work; group B (4/6 variables), use of Internet to cruise for sex partners, use of Internet to search for sexual health information, greater mobile phone usage, and early uptake of new technology; group C (2/8 variables), unprotected anal intercourse with unknown/discordant HIV status partner, and HIV negative status; group D (4/10 variables), last medical appointment >6 months ago or never, poorer satisfaction with health care services available, delayed/avoided testing because of privacy concern, and delayed/avoided testing because of access issue. Four interaction terms with age and nine with sexual orientation were included in the full multivariable model, as shown in Multimedia Appendix 3.

**Figure 1:**
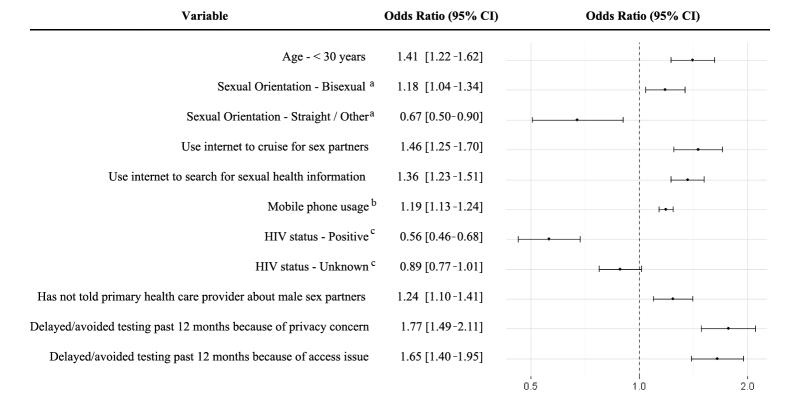
Correlates of intention to use Internet-based sexually transmitted infection testing selected by Bayesian Information Criterion in a survey sample of Canadian gay and bisexual men (N=7938). ^a^Referent group Sexual Orientation - Gay; ^b^Mobile phone usage measured on 3-point continuous scale; ^c^Referent group HIV status - negative.

The final model, as selected by BIC, is shown in Figure 1. Notably, of the nine variables positively associated with greater intention to use Internet-based testing in the final BIC model, three correspond to group B, Internet and technology usage: use Internet to cruise for sex partners (AOR 1.46, 95% CI 1.25-1.70), use Internet to search for sexual health information (AOR 1.36, 95% CI 1.23-1.51), and mobile phone usage (AOR 1.19, 95% CI 1.13-1.24). Three variables corresponded to group D, health care access: not “out” to primary health care provider (AOR 1.24, 95% CI 1.10-1.41), delayed/avoided testing due to privacy concerns (AOR 1.77, 95% CI 1.49-2.11), and delayed/avoided testing due to access issues (AOR 1.65, 95% CI 1.40- 1.95). No interaction terms were selected into the final BIC model.

### Perceived Benefits and Drawbacks of Service

The most frequent perceived benefits of the BC Internet-based STI/HIV testing program were greater privacy (2249/8388, 26.81%), convenience in general (1701/8388, 20.28%) and specifically, ability to get tested whenever (1048/8388, 12.49%). The greatest perceived drawbacks were the inability to see a doctor or nurse (1507/8388, 17.97%), wanting to talk to someone about results (1430/8388, 17.05%), not wanting results online (1084/8388, 12.92%), and low trust in the service generally (973/8388, 11.60%). The particular benefits and drawbacks perceived by survey respondents showed very little variation in sensitivity analyses (see Tables 1 and 2).

**Table 1:** Greatest perceived benefit to Internet-based STI and HIV testing among the survey sample of Canadian gay and bisexual men.

Benefit^a^	Total (N=8388)	Among those who intend to use service (n=5678)	Among those delaying/avoiding testing^b^ (n=4947)
	n (%)	n (%)	n (%)
Greater privacy	2249 (26.81)	1834 (32.30)	1551 (31.35)
Convenient	1701 (20.28)	1340 (23.60)	920 (18.60)
Get tested whenever	1048 (12.49)	755 (13.30)	596 (12.05)
No nurse/doctor	823 (9.81)	669 (11.78)	553 (11.18)
Save time	618 (7.37)	484 (8.52)	323 (6.53)
No waiting for app’t	448 (5.34)	301 (5.30)	249 (5.03)
No worry about running into someone you know	245 (2.92)	166 (2.92)	185 (3.74)
Other^c^	56 (0.67)	18 (0.32)	36 (0.73)
No particular benefit	1200 (14.31)	111 (1.95)	534 (10.79)

^a^Respondents were asked to choose one *greatest* benefit.

^b^Defined as those who reported no STI test in the last 12 months, OR any delay in testing in the last 12 months (for HIV-positive respondents); or no STI test AND no HIV test in the last 12 months, OR any delay in testing in the last 12 months (for HIV-negative/unknown status respondents).

^c^Not specified.

**Table 2:** Greatest perceived drawback to Internet-based STI and HIV testing among the survey sample of Canadian gay and bisexual men.

Drawback^a^	Total (N=8388)	Among those who do not intend to use service (n=2260)	Among those delaying/avoiding testing^b^ (n=4947)
	n (%)	n (%)	n (%)
Wouldn’t see doctor/nurse	1507 (17.97)	451 (19.96)	735 (14.86)
Want to talk to someone about results	1430 (17.05)	384 (16.99)	799 (16.15)
Don’t want results online	1084 (12.92)	407 (18.01)	652 (13.18)
Low trust in service	973 (11.60)	340 (15.04)	590 (11.93)
No printer	169 (2.01)	43 (1.90)	118 (2.39)
Other^c^	159 (1.90)	67 (2.96)	100 (2.02)
No particular drawback	3066 (36.55)	568 (25.13)	1953 (39.48)

^a^Respondents were asked to choose one greatest drawback.

^b^Defined as those who reported no STI test in the last 12 months, OR any delay in testing in the last 12 months (for HIV-positive respondents); or no STI test AND no HIV test in the last 12 months, OR any delay in testing in the last 12 months (for HIV-negative/unknown status respondents).

^c^Not specified.

## Discussion

### Principal Findings

Overall, we found that intention to use Internet-based testing for HIV and STI is high (5678/7938, 71.53%) and wide-ranging within this large online sample of gay and bisexual men in Canada, with little variation by participant characteristics. Our study suggests that Internet-based testing has the potential to reach nearly all subgroups of gay and bisexual men, including men at risk of STIs and HIV (as intention was 74.46% [1770/2377] among men reporting unprotected anal intercourse with an unknown or serodiscordant partner, and 73.70% [283/384] among men reporting an STI or hepatitis C diagnosis, in the past year) and facing current barriers to accessing testing (72.90% [2930/4019] among men not tested for HIV in the past year, and 83.2% [357/429] to 83.69% [944/1128] among men reporting delaying or avoiding testing in the past year because of privacy concerns, access issues, or distance to testing services).

On multivariable analysis, men who reported current barriers to accessing appropriate health care and STI/HIV testing were more likely to intend to use Internet-based testing (3/10 variables retained in the final model). In our sample, a large proportion of participants (4217/8388, 50.27%) reported not having disclosed to their primary health care provider that they were sexually active with men. Not being “out” to a primary care provider has been associated with undiagnosed HIV infection and less frequent rates of HIV testing [53,54]; we found greater intention to use Internet-based testing in this group, which may help to bridge this gap. Intent to use Internet-based testing was also more likely among the small but important proportion of participants in our sample who reported delaying or avoiding testing in the past 12 months because of privacy concerns (1140/8388, 13.59%) or because of access issues such as not knowing where to get a test or needing to wait for an appointment (1243/8388, 14.82%).

Men who identified as HIV positive (667/8388, 7.95% of our sample) demonstrated less intention to use Internet-based testing, which we postulate is related to adequate STI testing access through routine care, which confirms previous qualitative findings from our group [41], or a greater appeal of Internet-based testing for HIV testing among HIV negative men. However, men whose HIV status was unknown (1966/8388, 23.44%) also demonstrated less intention to use Internet-based testing after adjustment for other characteristics, which is concerning as this is a population of MSM who are not currently engaged in HIV testing. Unlike other studies, we did not observe an association with behavioral measures such as sexual risk or substance use, after adjusting for covariates [27,29,31,33,34,36]. Encouragingly, we did not find that sociodemographic variables such as ethnicity, income, education, and residence were influential on intention to use Internet-based testing, which differs from previously published studies in this field [27,31,32,37,38,40]. Within this large online sample of gay and bisexual men, Internet-based testing programs such as GetCheckedOnline may not exacerbate existing health inequities along these sociodemographic lines.

One reason for these differences may be that the characteristics of MSM that we considered allowed for better explanation of the variability in intention to use Internet-based testing. In addition to variables related to facing current barriers to access appropriate health care, we found variables related to Internet and technology use were most influential in our final model (3/6 retained): use of the Internet to cruise for sex partners or to search for health information, and mobile phone usage. Internet sex-seeking has long been a primary motivation for developing Internet-based sexual health interventions [55]; that a majority of MSM look for sex partners online underscores the importance of the Internet as a health service delivery venue (7430/8388, 88.58% in our online sample and typically over 50% of MSM in venue-based samples [56]). Given that use of the Internet to search for health information and use of mobile phones for more than phone calls were also associated with intention to use, our findings suggest that ease and facility with online technologies may be an important influence on uptake of Internet-based testing (ie, the “second-level” digital divide) [39]. This appears independent of the influence of age, which may be related to greater acceptability of online services by persons born in the era of digital technology compared to older persons [40]. While we found a consistent and significant gradient between diffusion of innovations and intention to use in our full model, this was not retained in the final model.

As hypothesized, we found that age and sexual orientation were influential on intention to use Internet-based testing and retained in the final model (greater intention among men <30 years; compared to gay men, bisexual men were more likely and straight/other men were less likely to intend to use Internet-based testing). The association with intention to use Internet-based testing varied across subgroups of men by age and sexual orientation. Notwithstanding the primary conclusion from our results—that intention to use Internet testing is high across varying subgroups of men—the statistical interactions described here suggest that where more nuanced decisions regarding service promotion and delivery are required, program planners must attend to the potentially different (sometimes opposite) intentions and needs of subgroups of gay and bisexual men, particularly those related to sexual orientation and identity.

The perceived benefits and drawbacks identified by Canadian gay and bisexual men in this study did not significantly differ in sensitivity analyses, and overall, men perceived more benefits than drawbacks: the percentages identifying no particular benefit or drawback were 14.31% (1200/8388) and 36.55% (3066/8388) respectively. The most common perceived benefits were greater privacy, convenience in general, and specifically, being able to test at any time; the most common perceived drawbacks were not seeing a doctor or nurse, not being able to talk to someone about the results, not wanting results online, or low trust in the service. These were also the most common perceived benefits and drawbacks from earlier focus groups focused on the GetCheckedOnline program model [41].

### Limitations

Our study had a number of limitations. These findings are not generalizable to all gay, bisexual, and other men who have sex with men in Canada, due to the online nature of this convenience sample, recruited primarily from sex-seeking websites. Furthermore, as participants were recruited for an online survey (and likely have some degree of ease with Internet use or predisposition to online services), we may have overestimated intention to use Internet-based testing, including potentially among some subgroups of interest (eg, MSM with poor Internet access, men of minority ethnicities where English may be a second language). However, the relative differences between subgroups within our sample may be accurate for all men who have sex with men in Canada. As an open-access survey, it is possible that multiple submissions from the same individual may have occurred; however, we believe this to be unlikely due to the lack of incentive and time required to complete the survey. As a self-completed survey, it is possible that recall bias may have affected responses to questions spanning the prior 12 months. Finally, while measuring intention to use a service is considered predictive of actual use, it will be important to measure actual diffusion and uptake of GetCheckedOnline among MSM once implemented.

### Conclusions

In summary, we observed high intention to use Internet-based testing among MSM in Canada given a brief description of the GetCheckedOnline program model. The high intention to use observed in our study appears most related to the perceived benefits of greater privacy, convenience, and being able to test at any time. Importantly, those who reported facing current barriers to appropriate health care and testing less frequently had higher intended use of Internet testing; intention to use was also more likely among individuals reporting greater use of technology. Our findings differ from that of other studies that have assessed the characteristics associated with acceptability of online sexual health services among youth or MSM (ie, little observed impact of sociodemographic or sexual risk characteristics). Our findings affirm that barriers to health care and technology use are important variables to consider in the design, implementation, and evaluation of online sexual health services. As we go forward, considering the requirements of less technology-savvy MSM and how the service could be more appealing to MSM of unknown HIV status will also be important.

## Multimedia Appendix 1

Definitions of all explanatory variables.

## Multimedia Appendix 2

Characteristics of survey sample of Canadian gay and bisexual men and intention to use Internet-based testing by key variables, across the four domain groups (N=8388).

## Multimedia Appendix 3

Interactions between explanatory variables with age and sexual orientation in the full model.

## Abbreviations

AIC: Akaike information criterion
AOR: adjusted odds ratio
BIC: Bayesian information criterion
HIV: human immunodeficiency virus
MSM: men who have sex with men
STI: sexually transmitted infection
