# Supplementary material for: Factors Associated With Intention to Use Internet-Based Testing for Sexually Transmitted Infections Among Men Who Have Sex With Men
Source: J Med Internet Res. 2013 Nov 14;15(11):e254. doi: 10.2196/jmir.2888 (PMC3841365; doi:10.2196/jmir.2888)
Supplement: Supplementary file 1 [file jmir_v15i11e254_app1.pdf]

Appendix 1.
Table: Definitions of all explanatory variables

A. Socio-demographic variables (n=14)

**Variable**	**Definition / Wording in questionnaire**	**Levels / Categories**	**Variable type (in analysis)**
Age	Age at time of survey, years	<30; ≥30 years	Categorical
Sexual orientation	"How do you see yourself…?"	Gay (homosexual); Bi (bisexual); Straight (heterosexual); Other (last 2 categories grouped for analysis)	Categorical
Gender of sex partners	"Sex in the last 12 months…"	No sex at all; sex with men only; sex with women only; sex with both men and women (last 2 categories grouped for analysis)	Categorical
Marital/partnership status	"Are you currently…?"	Single; married to a man; partnered with a man (preceding 2 categories grouped for analysis); married to a woman; partnered with a woman (preceding 2 categories grouped for analysis); separated, divorced from a man; separated, divorced from a woman (preceding 2 categories grouped with 'single' for analysis); other	Categorical
Living situation	"What is your living situation?"	Shared occupancy; sole owner/occupant; dormitory; couch surfing, home minding; no permanent address; other (last 4 categories grouped for analysis)	Categorical
Education	"What is the highest level of education that you have completed?"	Some high school; high school; some college/university (preceding 3 categories grouped for analysis); college; university; doctorate, PhD, MD, etc. (last 3 categories grouped for analysis)	Categorical
Ethnicity	"How do you describe yourself to other guys?"	African; Asian; Caribbean; Caucasian; First Nation; Inuit; Métis (preceding 3 categories grouped as 'Aboriginal' for analysis); Latino/Hispanic; Middle Eastern; Pacific Islander; South Asian; Mixed; Other (all ethnicities other than Caucasian, Asian, Aboriginal, and Latino grouped for analysis due to small	Categorical

Variable	Definition / Wording in questionnaire	Levels / Categories	Variable type (in analysis)
		sample size)	
Income	"What was your income in the last year?"	Under $10,000; $10,000-19,000; etc. to $99,999; $100,000+ (categorized as <$30K, $30-79K, ≥$80K for analysis)	Categorical
Province	"Where do you live?"	Listing of all 10 provinces; territories; outside Canada (territories, Saskatchewan, and Manitoba grouped for analysis; New Brunswick, Newfoundland and Labrador, Prince Edward Island, and Nova Scotia grouped as 'Atlantic'; outside Canada excluded from analysis)	Categorical
Environment	"What best describes your environment?"	Urban; suburban; rural; remote (preceding 2 categories grouped for analysis); other (excluded from analysis)	Categorical
Stage of "coming out" about sexuality with other guys	"How old were you when you 'came out' about your sexuality with other guys?" [with option: "never have come out about my sexuality"]	Those who selected "never have come out about my sexuality" labelled "has never 'come out'" for analysis	Categorical
"In general people in the area where I live are accepting of gay/bi men."	-	Definitely agree; somewhat agree; somewhat disagree; definitely disagree	Categorical
"Out" about sexuality at work	"How 'out' are you about your sexuality in your work environment (main income)?"	Everyone knows; some know (preceding 2 categories grouped as 'at least some know' for analysis); most don't know; no one knows (preceding 2 categories grouped as 'at least some know' for analysis); not applicable, not working, retired	Categorical
% of free time spent with other gay men	"How much of your free time do you usually spend hanging-out with other gay men?"	Little; 25%; 50%; 75%; most (last 3 categories grouped as '50% or more' for analysis)	Categorical

B. Internet and technology variables (n=6)

Variable	Definition / Wording in questionnaire	Levels / Categories	Variable type (in analysis)
Use internet to…	"In the last 12 months, have you used the	Cruise for sex partners;<br>Meet a potential boyfriend;	Categorical

**Variable**	**Definition / Wording in questionnaire**	**Levels / Categories**	**Variable type (in analysis)**
	internet to… ?"	Search for sexual health information	
Internet usage scale	"How often do you use the following internet sites or services?"	1-point each (cumulative) for regular/daily use of: Facebook; Twitter; Skype; Wikipedia; YouTube; Netflix; iTunes; Amazon	Continuous (8-point scale)
Mobile phone usage scale	"Do you use your cell phone for any of the following?"	1-point each (cumulative) for: Texting; surf the internet; use apps to navigate, entertain, play games, etc.	Continuous (3-point scale)
Uptake of new technology	"When do you usually buy new tech products after they become available (computer games, smart phone, 3D TV, etc.)?"	Amongst the first to own; early purchaser; when I see others have it; wait for the improved version; when almost everyone else has it	Categorical

C. Sexual risk variables (n=8)

**Variable**	**Definition / Wording in questionnaire**	**Levels / Categories**	**Variable type (in analysis)**
Sexually active with guys other than primary partner	"What are you into at the moment?"	Not sexually active; sex with primary partner only (preceding 2 categories grouped for analysis); hooking-up; dating; sex with my primary partner plus other guys; sex buddies; group sex; other (last 6 categories grouped for analysis)	Categorical
Number of sex partners, last 12 months	"In total, how many guys have you had sex with in the last 12 months?"	Numerical response; categorized *a priori* as: 0-1; 2-5; 6-10; 11-20; ≥21	Categorical
Sex in last 12 months perceived to be risky for HIV	"In the last 12 months, have you had any sex that you thought might have risked HIV transmission?"	Yes; no; not sure	Categorical
UAI with partner of unknown/discordant HIV status, last 12 months	"How many guys whose HIV status was unknown to you have you fucked without condoms?"; "How many guys whose HIV status was unknown to you have fucked you without condoms?"; "How many guys whose HIV status	none; 1; 2-5; 6-9; 10-19; 20+ (any response 1 or greater to any of the three questions was categorized as "yes" for analysis)	Categorical

Variable	Definition / Wording in questionnaire	Levels / Categories	Variable type (in analysis)
	was opposite yours have you fucked or have fucked you without condoms?”		
STI or HCV diagnosis, last 12 months	“Have you been diagnosed with any of the following?” [last 12 months]	Syphilis; gonorrhea; Chlamydia; herpes; genital warts; hepatitis C (HCV) (any response to these STI categorized as “yes” for analysis)	Categorical
HIV status	“What was your latest HIV test result?”	HIV-positive; HIV-negative; I’ve never had an HIV test (or result)	Categorical
Binge drinking	“How often do you consume more than 5 drinks in one sitting (beer, wine, and spirits)?”	Never; occasionally (preceding 2 categories grouped for analysis); some weekends; most weekends; some days and most weekends; most days (last 4 categories grouped for analysis)	Categorical
Party drug use	“How often have you used the following…?”	Cocaine; crystal; “E” ecstasy; “G” GHB; “K” ketamine (any ‘regular’ or ‘daily’ response to any one of these substances categorized as “yes” for analysis)	Categorical

D. Health care access variables (n=10)

Variable	Definition / Wording in questionnaire	Levels / Categories	Variable type (in analysis)
Routine medical care location	“Where do you usually get routine medical care?”	Family physician; walk-in medical clinic; emergency center (hospital); no routine care available; other	Categorical
Last medical appointment	“When was your last medical appointment?”	Within 6 months; 6 months – 1 year; 1-2 years; over 2 years ago; I’ve never had a medical appointment (last 4 categories grouped for analysis)	Categorical
Satisfaction with health care services available	“How satisfied are you with the health care services available to you?”	Very satisfied; satisfied; not very satisfied; not at all satisfied	Categorical
Told primary healthcare provider about being sexually active with men	“Have you told your primary health care provider that you have sex with other men?”	Yes; no; not sure (last 2 categories grouped for analysis)	Categorical
Ever dropped a provider because of anti-gay attitude	“Have you ever dropped a care provider due to his/her attitude (homo-negative, sexist, anti-gay)?”	Yes; no; not sure (last 2 categories grouped for analysis)	Categorical

**Variable**	**Definition / Wording in questionnaire**	**Levels / Categories**	**Variable type (in analysis)**
STI/HIV test in last 12 months	"Have you been tested within the past 12 months?"	STI testing (yes/no); HIV testing (yes/no)	Categorical
Delayed/avoided testing in past 12 months because of…	"Have any of the following caused you to delay, avoid, or skip testing within the past 12 months?"	Didn't know where to go; needed an appointment; wait was too long; clinic wasn't open when I could test (preceding 4 categories grouped as 'access issues' for analysis); clinic was too far away; didn't want to see a doctor or nurse; couldn't get anonymous testing (preceding 2 categories grouped as 'privacy concern' for analysis); don't like needles; other; none of above (last 3 categories not used in analysis)	Categorical
