# Supplementary material for: Factors Associated With Intention to Use Internet-Based Testing for Sexually Transmitted Infections Among Men Who Have Sex With Men
Source: J Med Internet Res. 2013 Nov 14;15(11):e254. doi: 10.2196/jmir.2888 (PMC3841365; doi:10.2196/jmir.2888)
Supplement: Supplementary file 2 [file jmir_v15i11e254_app2.pdf]

Appendix 2.

Table: Characteristics of survey sample of Canadian gay and bisexual men and intention to use Internet-based testing by key variables, across four domain groups: **A)** socio-demographics; **B)** Internet and technology usage; **C)** STI/HIV and risk; and **D)** health care access (N=8388)

A. Sociodemographics

**Characteristic**	**Level**	**n (%), or median (IQR)**	**% intend to use service**	**UOR (95% CI)** N=7938	**Full Model**[a] **AOR (95% CI)** N=7873
Age	≥30 years	6520 (77.7)	69.0	REF	[b]
	<30 years	1868 (22.3)	80.0	**1.80 (1.58-2.04)**	
Sexual orientation	Gay	5410 (64.5)	70.1	REF	[c]
	Bisexual	2719 (32.4)	74.8	**1.26 (1.14-1.41)**	
	Straight/Other	259 (3.1)	66.9	0.86 (0.65-1.14)	
Gender of sex partners	Men only	5955 (71.0)	70.8	REF	[d]
	Women (any degree)	2018 (24.1)	73.7	**1.15 (1.03-1.30)**	
	No sex	415 (4.9)	70.6	0.99 (0.78-1.25)	
Marital/partnership status	Single	4223 (50.3)	72.0	REF	[d]
	Partnered to man	2216 (26.4)	68.3	**0.84 (0.75-0.94)**	
	Partnered to woman	1805 (21.5)	74.4	1.13 (1.00-1.29)	
	Other	144 (1.7)	72.1	1.01 (0.68-1.49)	
Living situation	Sole occupant	3701 (44.1)	70.3	REF	REF
	Shared occupancy	4350 (51.9)	72.1	1.09 (0.99-1.21)	0.99 (0.88-1.1)
	No permanent residence	337 (4.0)	77.7	**1.48 (1.12-1.94)**	1.18 (0.87-1.6)
Education	College/university degree	4792 (57.1)	70.5	REF	REF
	High school/some college	3596 (42.9)	73.0	**1.13 (1.02-1.25)**	1.11 (0.99-1.24)
Ethnicity	Caucasian	7313 (87.2)	71.4	REF	REF
	Asian	212 (2.5)	70.8	0.97 (0.71-1.32)	0.75 (0.53-1.04)
	Aboriginal	169 (2.0)	71.7	1.02 (0.71-1.45)	0.93 (0.63-1.35)
	Latino	112 (1.3)	80.7	**1.68 (1.04-2.71)**	1.60 (0.96-2.66)
	Other	582 (6.9)	71.6	1.01 (0.83-1.23)	0.85 (0.69-1.05)
Annual Income	<$30K	2394 (28.5)	72.4	REF	REF
	$30-79K	4155 (49.5)	71.1	0.94 (0.83-1.05)	1.10 (0.92-1.32)
	≥$80K	1839 (21.9)	71.4	0.95 (0.83-1.09)	0.98 (0.78-1.23)
Province of residence	Prairies/Territories	654 (7.8)	67.4	REF	REF
	British Columbia	1805 (21.5)	69.4	1.10 (0.90-1.34)	1.19 (0.97-1.48)
	Alberta	1065 (12.7)	74.5	**1.41 (1.14-1.76)**	**1.27 (1.01-1.60)**
	Ontario	3368 (40.2)	71.6	**1.22 (1.02-1.47)**	**1.24 (1.02-1.51)**

Characteristic	Level	n (%), or median (IQR)	% intend to use service	UOR (95% CI) N=7938	Full Model [a] AOR (95% CI) N=7873
	Quebec	1049 (12.5)	72.0	1.25 (1.00-1.55)	**1.36 (1.08-1.73)**
	Atlantic	447 (5.3)	77.0	**1.62 (1.22-2.14)**	**1.57 (1.17-2.11)**
Living environment[e]	Urban	4897 (58.4)	70.7	REF	[b]
	Suburban	2214 (26.4)	73.9	**1.17 (1.04-1.31)**	
	Rural/remote	1245 (14.8)	70.2	0.98 (0.85-1.12)	
Stage of "coming out"	Already "out"	5295 (63.1)	70.1	REF	REF
	Has never "come out"	3093 (36.9)	74.0	**1.21 (1.10-1.34)**	1.05 (0.89-1.25)
"In general, people in the area where I live are accepting of gay/bi men."	Definitely agree	2358 (28.1)	69.0	REF	REF
	Somewhat agree	4005 (47.7)	71.8	**1.14 (1.02-1.28)**	1.00 (0.88-1.14)
	Somewhat disagree	1293 (15.4)	76.1	**1.43 (1.22-1.67)**	1.17 (0.97-1.40)
	Definitely disagree	732 (8.7)	70.0	1.05 (0.87-1.26)	0.81 (0.64-1.01)
"Out" about sexuality at work	At least some know	3881 (46.3)	69.0	REF	[b]
	Few or no people know	3690 (44.0)	75.1	**1.35 (1.22-1.5)**	
	Not applicable	817 (9.7)	66.7	0.9 (0.76-1.06)	
% of free time spent with other gay men	50% or more	1867 (22.3)	70.5	REF	REF
	<50%	6521 (77.7)	71.8	1.06 (0.95-1.20)	0.99 (0.87-1.14)

B. Internet and technology use

Characteristic	Level	n (%), or median (IQR)	% intend to use service	UOR (95% CI) N=7938	Full Model[a] AOR (95% CI) N=7873
Use Internet to cruise for sex partners	No	958 (11.4)	63.5	REF	REF
	Yes	7430 (88.6)	72.5	**1.51 (1.31-1.76)**	**1.39 (1.15-1.68)**
Use Internet to meet potential boyfriend	No	4227 (50.4)	69.5	REF	REF
	Yes	4161 (49.6)	73.5	**1.22 (1.1-1.34)**	1.06 (0.95-1.18)
Use Internet to search for sexual health information	No	2968 (35.4)	66.1	REF	[b]
	Yes	5420 (64.6)	74.5	**1.5 (1.36-1.66)**	
Internet usage scale (0-8 continuous scale)		2 (0, 3)		**1.12 (1.09-1.15)**	1.03 (0.99-1.07)
Mobile phone usage scale (0-3 continuous scale)		2 (1, 3)		**1.26 (1.21-1.32)**	**1.14 (1.08-1.20)**
Uptake of new technology (eg, smart phone)	Wait for everyone else	3312 (39.5)	66.0	REF	[b]
	Wait for improved version	2962 (35.3)	73.8	**1.45 (1.3-1.62)**	

	Buy it when others have it	594 (7.1)	74.3	**1.49 (1.22-1.83)**	
	Early purchaser	1166 (13.9)	77.3	**1.75 (1.49-2.05)**	
	Amongst the first to own	354 (4.2)	79.9	**2.05 (1.55-2.70)**	

C. STI/HIV and risk

Characteristic	Level	n (%), or median (IQR)	% intend to use service	UOR (95% CI) N=7938	Full Model[a] AOR (95% CI) N=7873
Sexually active with guys other than primary partner	No	1685 (20.1)	66.6	REF	REF
	Yes	6703 (79.9)	72.7	**1.33 (1.18-1.5)**	1.14 (0.89-1.33)
Number of sex partners, last 12 months [e]	0-1	1592 (19)	69.3	REF	REF
	2-5	2829 (33.7)	71.9	1.14 (0.99-1.31)	0.94 (0.80-1.11)
	6-10	1566 (18.7)	73.2	**1.21 (1.03-1.42)**	0.99 (0.81-1.20)
	11-20	1157 (13.8)	74.0	**1.26 (1.06-1.50)**	1.04 (0.83-1.29)
	≥21	1204 (14.4)	69.3	1.00 (0.85-1.19)	0.86 (0.69-1.07)
Sex in last 12 months perceived to be risk for HIV	No	5719 (68.2)	70.3	REF	REF
	Yes	1793 (21.4)	73.7	**1.18 (1.05-1.34)**	0.98 (0.83-1.14)
	Not sure	876 (10.4)	75.1	**1.27 (1.08-1.50)**	1.08 (0.89-1.30)
UAI with partner of unknown or discordant HIV status, last 12 mo	No	5871 (70.0)	70.3	REF	REF
	Yes	2517 (30.0)	74.5	**1.23 (1.11-1.37)**	**1.2 (1.04-1.38)**
STI or HCV diagnosis, last 12 mo	No	7974 (95.1)	71.4	REF	REF
	Yes	414 (4.9)	73.7	1.12 (0.89-1.41)	1.05 (0.81-1.35)
HIV status	Negative	5755 (68.6)	72.4	REF	[c]
	Positive	667 (8.0)	55.3	**0.47 (0.39-0.57)**	
	Never tested	1966 (23.4)	73.1	1.03 (0.92-1.16)	
Binge drinking (>5 drinks in one sitting)	Never or occasional	5901 (70.4)	70.5	REF	REF
	Some weekends or more often	2487 (29.6)	74.0	**1.19 (1.07-1.33)**	1.04 (0.93-1.17)
Party drug use (cocaine, crystal, ecstasy, GHB, or ketamine)	Never or occasional	8215 (97.9)	71.6	REF	[b]
	Regular or daily	173 (2.1)	66.0	0.77 (0.55-1.07)	

D. Health care access

Characteristic	Level	n (%), or median (IQR)	% intend to use service	UOR (95% CI) N=7938	Full Model[a] AOR (95% CI) N=7873
Routine medical	Family physician	6291 (75)	70.5	REF	[c]

Characteristic	Level	n (%), or median (IQR)	% intend to use service	UOR (95% CI) N=7938	Full Model[a] AOR (95% CI) N=7873
care location	Walk-in clinic	1614 (19.2)	75.1	**1.27 (1.11-1.44)**	
	Emergency room/hospital	139 (1.7)	68.4	0.91 (0.63-1.31)	
	No routine medical care available	217 (2.6)	77.1	**1.41 (1.02-1.96)**	
	Other	127 (1.5)	69.1	0.94 (0.62-1.41)	
Last medical appointment	Within past 6 months	5243 (62.5)	69.6	REF	[b]
	>6 months ago or never	3145 (37.5)	74.7	**1.29 (1.17-1.43)**	
Satisfaction with health care services available	Very satisfied	3143 (37.5)	67.0	REF	[b]
	Satisfied	3666 (43.7)	72.6	**1.31 (1.17-1.45)**	
	Not very satisfied	1205 (14.4)	76.7	**1.62 (1.39-1.90)**	
	Not satisfied at all	374 (4.5)	80.3	**2.01 (1.53-2.63)**	
Told primary health care provider about being sexually active with men	"Out" to provider	4171 (49.7)	68.1	REF	[c]
	Not "out" or unsure	4217 (50.3)	74.9	**1.40 (1.27-1.54)**	
Ever dropped a provider because of anti-gay attitude	No	7491 (89.3)	71.6	REF	REF
	Yes	897 (10.7)	70.7	0.96 (0.82-1.12)	1.02 (0.86-1.22)
STI test	Within past 12 months	4074 (48.6)	70.3	REF	[b]
	>12 months ago or never	4314 (51.4)	72.7	**1.13 (1.02-1.24)**	
HIV test	Within past 12 months	4093 (48.8)	70.1	REF	REF
	>12 months ago or never	4295 (51.2)	72.9	**1.15 (1.04-1.26)**	1.17 (0.97-1.40)
Delayed/avoided testing in past 12 months because of privacy concern	No	7248 (86.4)	69.5	REF	REF
	Yes	1140 (13.6)	83.7	**2.25 (1.91-2.66)**	**1.61 (1.34-1.93)**
Delayed/avoided testing in past 12 months because of access issue	No	7145 (85.2)	69.4	REF	REF
	Yes	1243 (14.8)	83.3	**2.21 (1.88-2.59)**	**1.6 (1.33-1.91)**
Delayed/avoided testing in past 12 months because too far from clinic	No	7952 (94.8)	70.9	REF	REF
	Yes	436 (5.2)	83.2	**2.04 (1.58-2.64)**	1.09 (0.82-1.45)

Notes:
[a] n=450 respondents who indicated "not applicable" to outcome (intention to use Internet-based testing) were excluded from regression models.

[b] Interaction between variable and sexual orientation (AOR stratified by sexual orientation presented separately in Table 2).
[c] Interaction between variable and age (AOR stratified by age presented separately in Table 2).
[d] Variable not included in multivariable model due to collinearity with covariates.
[e] Excluding non-applicable or blank responses (n=29 for living environment; n=36 for number of sex partners).
