# Supplementary material for: Factors Associated With Intention to Use Internet-Based Testing for Sexually Transmitted Infections Among Men Who Have Sex With Men
Source: J Med Internet Res. 2013 Nov 14;15(11):e254. doi: 10.2196/jmir.2888 (PMC3841365; doi:10.2196/jmir.2888)
Supplement: Supplementary file 3 [file jmir_v15i11e254_app3.pdf]

Appendix 3.
Table: Interactions between explanatory variables with age and sexual orientation in the full model

A. Interactions with age (<30, ≥30)

Characteristic	Level	UOR (95% CI) N=7938	Age < 30 years [a] AOR (95% CI) N=1794	Age ≥ 30 years [a] AOR (95% CI) N=6079
Sexual orientation	Gay	REF	REF	REF
	Bisexual	**1.26 (1.14-1.41)**	1.33 (0.6-2.98)	**1.94 (1.23-3.08)**
	Straight/Other	0.86 (0.65-1.14)	0.55 (0.08-3.85)	0.34 (0.10-1.10)
HIV status	Negative	REF	REF	REF
	Positive	**0.47 (0.39-0.57)**	1.18 (0.37-3.8)	**0.56 (0.45-0.71)**
	Never tested	1.03 (0.92-1.16)	1.37 (0.82-2.29)	**0.69 (0.58-0.82)**
Routine medical care location	Family physician	REF	REF	REF
	Walk-in clinic	**1.27 (1.11-1.44)**	0.93 (0.57-1.51)	0.85 (0.71-1.01)
	Emergency room/hospital	0.91 (0.63-1.31)	0.33 (0.09-1.25)	0.91 (0.57-1.47)
	No routine medical care available	**1.41 (1.02-1.96)**	1.49 (0.44-5.08)	**0.63 (0.41-0.97)**
	Other	0.94 (0.62-1.41)	0.62 (0.13-2.89)	1.12 (0.68-1.83)

B. Interactions with sexual orientation (gay, bisexual)

Characteristic	Level	UOR (95% CI) N=7938	Gay Men [a] AOR (95% CI) N=5066	Bisexual Men [a] AOR (95% CI) N=2574
Age	≥30 years	REF	REF	REF
	<30 years	**1.80 (1.58-2.04)**	**1.53 (1.17-1.99)**	1.05 (0.57-1.92)
Living environment[b]	Urban	REF	REF	REF
	Suburban	**1.17 (1.04-1.31)**	1.08 (0.92-1.27)	0.87 (0.57-1.34)
	Rural/remote	0.98 (0.85-1.12)	1.07 (0.88-1.31)	0.82 (0.49-1.37)
"Out" about sexuality at work	At least some know	REF	REF	REF
	Few or no people know	**1.35 (1.22-1.5)**	**1.32 (1.11-1.58)**	0.87 (0.49-1.54)
	Not applicable	0.90 (0.76-1.06)	1.18 (0.94-1.48)	0.78 (0.37-1.63)
Use Internet to search for sexual health information	No	REF	REF	REF
	Yes	**1.5 (1.36-1.66)**	**1.25 (1.09-1.44)**	**1.61 (1.11-2.34)**
Uptake of new technology (eg, smart phone)	Wait for everyone else	REF	REF	REF
	Wait for improved version	**1.45 (1.3-1.62)**	**1.19 (1.03-1.39)**	1.28 (0.85-1.94)

Characteristic	Level	**UOR (95% CI)** N=7938	**Gay Men [a] AOR (95% CI)** N=5066	**Bisexual Men [a] AOR (95% CI)** N=2574
	Buy it when others have it	**1.49 (1.22-1.83)**	**1.39 (1.06-1.84)**	0.95 (0.46-1.96)
	Early purchaser	**1.75 (1.49-2.05)**	**1.44 (1.16-1.79)**	1.18 (0.66-2.11)
	Amongst the first to own	**2.05 (1.55-2.7)**	**1.79 (1.25-2.55)**	2.07 (0.74-5.82)
Party drug use (cocaine, crystal, ecstasy, GHB, or ketamine)	Never or occasional	REF	REF	REF
	Regular or daily	0.77 (0.55-1.07)	1.05 (0.68-1.64)	0.40 (0.12-1.4)
Last medical appointment	Within past 6 months	REF	REF	REF
	>6 months ago or never	**1.29 (1.17-1.43)**	**1.18 (1.02-1.36)**	0.98 (0.67-1.45)
Satisfaction with health care services available	Very satisfied	REF	REF	REF
	Satisfied	**1.31 (1.17-1.45)**	1.11 (0.97-1.29)	1.17 (0.79-1.74)
	Not very satisfied	**1.62 (1.39-1.9)**	1.15 (0.93-1.42)	1.80 (1.00-3.24)
	Not satisfied at all	**2.01 (1.53-2.63)**	**1.81 (1.24-2.63)**	1.41 (0.53-3.77)
STI test	Within past 12 months	REF	REF	REF
	>12 months ago or never	**1.13 (1.02-1.24)**	1.07 (0.88-1.3)	0.81 (0.52-1.26)

Notes:
[a] Stratified odds ratios presented for variables with statistically significant (p<0.15) first-level interactions with sexual orientation or age. n=450 respondents who indicated "not applicable" to outcome (intention to use Internet-based testing) were excluded from regression models.
[b] Excluding non-applicable or blank responses (n=29 for living environment).
